# Turn It Off: An Action Research Study of Top Management Influence on Energy Conservation in the Workplace

**DOI:** 10.3389/fpsyg.2016.00389

**Published:** 2016-03-21

**Authors:** Sally V. Russell, Alice Evans, Kelly S. Fielding, Christopher Hill

**Affiliations:** ^1^Sustainability Research Institute, School of Earth and Environment, University of LeedsLeeds, UK; ^2^Institute for Teaching and Learning Innovation, The University of QueenslandBrisbane, QLD, Australia; ^3^School of Communication and Arts, The University of QueenslandBrisbane, QLD, Australia; ^4^Mater Health ServicesBrisbane, QLD, Australia

**Keywords:** energy conservation, pro-environmental behavior, workplace, sustainability, intervention, organizational culture, top management

## Abstract

This paper reports the results of an intervention study that aimed to encourage workplace energy conservation behavior by office-based employees. Taking a co-production approach we worked with the participating organization to design and implement an intervention that used the influence of top management commitment and prompts to encourage workplace energy reduction. Whilst past research has shown top management is related to workplace pro-environmental behavior, this study extends this work by examining a field-based intervention over a longitudinal period. The efficacy of the intervention was measured using observational and self-reported data over a period of 6 months. Results showed that there were significant changes in objective and self-reported energy conservation behavior, perceived top management commitment, organizational culture, norms, and knowledge regarding energy conservation behavior over the course of the study. The findings also demonstrated that the intervention was most successful for those behaviors where employees have individual responsibility. Implications for future research and practice are discussed.

## Introduction

Businesses are facing increasing pressure to reduce their electricity use as issues of climate change and limited resources become more prominent (De Young, 1993; Stern, 2000). While research to date has tended to focus on organizational policy and strategy as an effective mechanism to change organizational behavior, recent research has identified the many opportunities for gains to be made by looking inside the organization (Robertson and Barling, 2013; Unsworth et al., 2013; Young et al., 2015). One method of reducing electricity use is by encouraging employees to change their behavior in the workplace. Indeed, the role of employee behavior change in reducing the energy use of organizations cannot be understated. Research has shown that interventions can be quite effective at changing individual behavior (De Young, 1993), however, this finding has not been tested to the same extent in the workplace (Ones and Dilchert, 2012; Young et al., 2015). We argue that more research is necessary to examine whether these types of interventions are effective in the workplace. The findings from such an investigation have the potential to result in significant reductions in the use of electricity by organizations.

Organizations are large consumers of electricity and they are therefore under significant pressure to play a part in reducing electricity demand. For example, two thirds of electricity produced in Australia is supplied to commercial customers. Over the period from 2008–2009 to 2010–2011, the use of electricity by industrial consumers increased by 18% (Australian Bureau of Statistics, 2013). These figures also highlight the opportunity for businesses to reduce costs by reducing electricity use.

In this article we report the results of an intervention study conducted at a large Australian hospital with the goal of encouraging energy conservation behavior for office-based employees. Drawing on literature from organizational and environmental psychology we took an action research approach in the design and implementation of the workplace pro-environmental behavior change intervention.

Research has shown that the determinants of behavior in the workplace are different to those in other contexts such as households (Andersson and Bateman, 2000; Steg and Vlek, 2009). For example, within the household individual behavior to reduce energy can be directly linked to saving money on bills. In the workplace, however, this link is much more distant and the relevance of self-interested monetary savings becomes much less relevant for individual employees (Griskevicius et al., 2012). As such, strategies that are effective in encouraging households to engage in more pro-environmental behavior may be different to those that are effective in a workplace context.

There is some evidence to suggest that employees look to the organization and its managers to provide cues as to how to behave in relation to environmental issues (Andersson and Bateman, 2000; Ramus and Steger, 2000; Young et al., 2015), yet further research is needed to investigate this issue. In particular, we argue that the influence of top management commitment on employee pro-environmental behavior is an area that deserves further attention (Morgeson et al., 2013; Jones Christensen et al., 2014). Past research has demonstrated that top management support is significantly correlated with pro-environmental behavior (Ramus and Steger, 2000; Robertson and Barling, 2013), however, there are far fewer studies that go beyond correlational analysis (Vlachos et al., 2013). In this research study we aim to contribute to this burgeoning area by testing the influence of top management commitment in a field-based intervention study.

Furthermore, the current study aims to contribute a practical understanding of how psychological interventions can be used to promote more pro-environmental behavior in a workplace setting. While interventions are often tested in laboratory settings with strict controls, we note that applied studies such as the one reported here have received much less research attention despite researchers highlighting this as important step in understanding pro-environmental behavior in the workplace (Andersson et al., 2013; Unsworth et al., 2013).

The action research approach, or what could also be termed co-production, entails participation by a group of people who work together to co-create positive outcomes for both parties (McNiff and Whitehead, 2001; Thompson and Perry, 2004). Businesses and researchers are increasingly using action research (McNiff and Whitehead, 2001) and co-creation (Chen et al., 2012) to bring together knowledge from researchers and practitioners in order to have a positive influence on organizational practice. In this study we take the approach of co-production with a social value (Guston, 2001; Sanders and Simons, 2009), and aim to test the efficacy of an initiative designed to improve the environmental performance of an organization using behavior change techniques.

In the following sections of this article we first review research that examines workplace behavior and identify the importance of the organizational variables of top management commitment and organizational culture in determining workplace pro-environmental behavior. We then introduce the behavior change theories that were used to design the intervention, with a particular focus on the use of persuasive techniques. The proceeding sections of the article outline the methods and results, and then in the final section we discuss the implications of our research for both theory and practice.

## Background and Theoretical Framework

As noted by Andersson et al. (2013), to date there has been a lack of emphasis on the contribution that organizational scholars can make to research on workplace pro-environmental behavior. Workplace pro-environmental behavior can be defined as “any action taken by employees that she or he thought would improve the environmental performance of the company” (Ramus and Steger, 2000, p. 606). Past research has been dominated by studies that have examined individual factors that affect pro-environmental behavior. Most notably, the individual variables of attitudes, norms, and knowledge have been shown to be key determinants of workplace pro-environmental behavior (Young et al., 2015). An attitude reflects a person’s positive or negative evaluation of an attitude object (Ajzen, 1991) and refers to how favorable individuals feel about engaging in a particular behavior (Ajzen, 1991). A positive attitude toward a workplace pro-environmental behavior should result in more engagement in this type of behavior. Similarly, subjective norms – perceived social pressure to engage in certain behaviors – can also result in greater performance of the target behavior (Ajzen, 1991; Rivis and Sheeran, 2003). Rivis and Sheeran (2003) conducted a meta-analysis and found that descriptive norms are also predictive of behavior. Descriptive norms differ from subjective norms in that they refer to perceptions of what other people typically do (Cialdini, 2003), rather than the perceived social pressure evidenced in subjective norms. Finally, knowledge about a particular behavior is also an important individual variable, because in order to perform the behavior one must first know how to do so (Abrahamse et al., 2005).

While environmental psychology has shown that these variables have a strong influence on individuals, within the workplace, organizational factors also become important. Young et al. (2015) showed that perceptions of the organization and its leaders also play an important role in explaining workplace pro-environmental behavior. In particular, they found that organizational variables of top management support and organizational culture are key in understanding how employees behave in relation to environmental issues (Tudor et al., 2008; Young et al., 2015).

As noted by Young et al. (2015), organizational culture is important in determining workplace pro-environmental behavior. In this context the environmental culture of the organization can be considered the degree to which environmental issues are considered in the goals, values, and day-to-day operation of the company (Banerjee et al., 2003). Top management also play an important role in organizational culture in that their level of involvement in sustainability is a clear demonstration of the depth of the sustainability culture within the organization (Russell and McIntosh, 2011).

According to Banerjee et al. (2003, p. 110), “top management demonstrates its commitment to environmentalism by appointing senior managers responsible for overseeing the firm’s environmental orientation and strategies.” Management support can be as simple as making a written commitment to improve the organization’s sustainability (Zibarras and Ballinger, 2011), although Ramus and Steger (2000) found it could be more than just a written commitment. Encouraging environmental innovation, competence building, communication, reward and recognition, and management of goals and responsibilities are all important aspects of top management commitment (Ramus and Steger, 2000). These types of encouragement have all been found to positively influence employee environmental initiatives (Ramus and Steger, 2000). For the purposes of our research we define management support as ‘top management commitment,’ or the extent to which top management is perceived to be supportive of pro-environmental behavior in the workplace (Banerjee et al., 2003).

Leadership literature also has much to offer in understanding how employees can be encouraged to engage in more pro-environmental behavior (e.g., Jones Christensen et al., 2014). In particular the concept of environmentally specific transformational leadership has been demonstrated to have a significant effect (Robertson and Barling, 2013). Transformational leaders often use idealized influence and thereby become role models for employees; that is they do “what’s right rather than what is expedient” (p. 178). In this way the most effective leaders engage in modeling behavior and influence their employees to engage in actions that reduce the organization’s impact on the natural environment. In this study we aim to test this theory in order to go beyond past findings that have largely been correlational in nature.

Research by Ramus and Steger (2000), for example, showed that strong signals of top management support were correlated with increases in employee implementation of workplace pro-environmental behavior. Similarly, Robertson and Barling (2013) have shown that transformational leadership characteristics are associated with greater self-reported employee pro-environmental behavior. Taken together these studies point to the importance of top management support for workplace pro-environmental behavior.

While these studies have been instrumental in recognizing the importance of top management support, they are limited in two ways. First, these studies are correlational and therefore it is not possible to examine how or whether top management commitment can influence employee pro-environmental behavior over time. Additionally, these studies rely on self-reported rather than observational measures of pro-environmental behavior, a limitation of much of the intervention literature (Young et al., 2015). In this research we aimed to further understanding by examining whether an intervention designed to emphasize top management support could be used to encourage employees to engage in more workplace pro-environmental behavior. Furthermore, we aimed to use observational measures of behavior and to test our intervention in a field setting.

### Behavior Change Theory

In the current study the intervention was designed with a focus on emphasizing top management commitment in order to encourage staff to engage in more pro-environmental behavior within the workplace. This was achieved using the behavior change techniques of modeling and prompts. Evidence suggests that using more than one strategy can greatly increase the effectiveness of the intervention (Abrahamse et al., 2005), and therefore these two key strategies were considered to be particularly appropriate in the organizational context.

The first approach, modeling, is consistent with the idealized influence of transformational leadership (Robertson and Barling, 2013). Research has shown that the effect of top management commitment can be enhanced by ensuring that managers demonstrate or model the actions that they are trying to encourage in their staff (Feasby and Wells, 2011; Kane, 2011). Seeing management demonstrate or model the target behavior in the workplace goes above and beyond simply making a commitment to improve sustainability, because it shows that management are actually following through on their commitments. If the management team is not engaging in the behavior in the workplace then employees may not feel that they should. For instance, Blok et al. (2015) showed that leadership support and exemplary pro-environmental behavior by leaders are important factors when it comes to pro-environmental behavior in the workplace. They found that when managers were seen to display pro-environmental behavior themselves, this had a significant positive impact on employee’s intention to act pro-environmentally.

The modeling approach is also consistent with the provision of influence using social norms. Social norms are the implied rules about how to act or the accepted ways of doing things (Turner, 1991). Social psychological research has a long history that demonstrates how norms have been used to influence behavior, by encouraging individuals to engage in behaviors they observe in others and feeling a sense of pressure to conform (Schultz et al., 2008; Goldstein et al., 2011). Research has shown that social norm messages have been more effective in changing pro-environmental behavior than other types of persuasive messages that try and change behavior by appealing to environmental protection norms or financial goals (Goldstein et al., 2008; Nolan et al., 2008; Schultz et al., 2008).

Persuasive messages have been shown to be effective in changing behavior, however, one of the key limitations is the extent to which such changes endure over the long term (Goldstein et al., 2011). Thus, one of the key issues with workplace pro-environmental behavior is how to effectively encourage employees to engage in such behavior, and to make it part of their everyday routine over the long term. Prompts have been shown to be one way to encourage behavior, and they can be defined as “a visual or auditory aid that reminds us to carry out an activity that we might otherwise forget” (McKenzie-Mohr and Smith, 1999, p. 61). In other words, prompts are an effective way of reminding individuals about new tasks until they become established as routines. Even after a task has become routine, prompts can continue to be useful by helping to maintain these routines.

Prompts are one of the most simple and least expensive behavior change interventions (Schultz et al., 1995), and they have been applied to various contexts and behaviors including pro-environmental behavior (Lehman and Geller, 2004). In addition to being inexpensive and simple, prompts are also less intrusive compared to other strategies such as social pressure and material disincentives (De Young, 1993; Schultz et al., 1995). Further, they can produce immediate changes in behavior, and can potentially influence large numbers of people (De Young, 1993). Moreover, there are many studies, reviews, and meta-analyses that conclude that prompts are an effective tool for increasing pro-environmental behavior (Schultz et al., 1995; Lehman and Geller, 2004; Osbaldiston, 2004; Osbaldiston and Schott, 2012).

Osbaldiston’s (2004) meta-analysis showed that prompts have been used successfully to increase recycling, water conservation, and energy conservation. In particular, using prompts was the second most effective method for increasing energy conservation behavior, second only to the more expensive strategy of providing incentives (Osbaldiston, 2004). A more recent meta-analysis by Osbaldiston and Schott (2012) supported the earlier findings, and concluded that prompts are one of the most effective ways to increase pro-environmental behavior. Lehman and Geller (2004) provided a summary of studies on prompts and concluded that prompts are particularly effective when targeting littering, energy use, and recycling.

A research study reported by Sussman and Gifford (2012) is one of very few studies on prompts within a workplace setting. Their study used prompts to encourage people to turn off the lights in unoccupied public bathrooms at a university campus. The prompts were effective at changing behavior: compared to bathrooms without prompts, bathrooms with prompts were eight times more likely to have lights turned off (Sussman and Gifford, 2012).

Using a combination of intervention techniques as described above, we worked with an organization to design an intervention to create enduring positive change in workplace pro-environmental behavior. In testing the efficacy of the intervention, our overarching hypothesis of the study is,

Hypothesis: Enhancing the visibility of top management commitment through modeling and prompts will increase pro-environmental behavior in the workplace.

### Behavioral Focus

The purpose of this research is to investigate the effectiveness of an intervention that aimed to demonstrate top management commitment and in doing so encourage workplace pro-environmental behavior. Research suggests that a focus on a narrow set of targeted behavior can improve the effectiveness of intervention design (Abrahamse et al., 2007). We therefore targeted specific behaviors and narrowed the focus of our research to workplace electricity conservation, which we define as any actions by employees that reduce the electricity consumed by the organization. Our overarching aim was to investigate the efficacy of an intervention to encourage energy conservation behavior in the workplace.

From our review of the literature it is clear that top management commitment plays an important role in determining workplace pro-environmental behavior (Russell et al., 2007; Russell and McIntosh, 2011; Young et al., 2015). We argue that an intervention designed to enhance the visibility of top management support will therefore result in an increase in workplace energy conservation behavior. Thus, our aim was to examine the efficacy of an intervention approach that enhances the visibility of top management commitment to energy conservation behavior in the workplace.

## Materials and Methods

**FIGURE 1:**
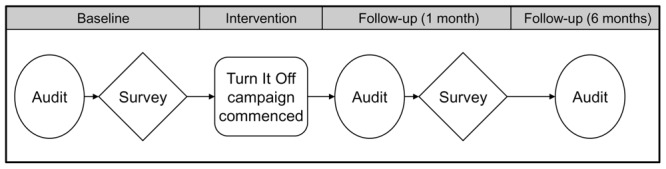
**Overview of the Turn It Off project**.

The current study consisted of three main components: the intervention (the “Turn It Off” campaign), energy audits, and surveys. **Figure 1** depicts the overall timeline of the project, and the pre-test post-test design. This study was carried out in accordance with the recommendations of the Australian National Statement on Ethical Conduct in Human Research. All participants gave informed consent to participate in the study.

### Intervention Design and Administration

The purpose of the intervention was to encourage energy conservation behavior in the workplace through the use of persuasive techniques emphasizing top management. The energy saving behaviors that were targeted were selected by the organizational representatives and included turning off lights in unoccupied offices, unplugging chargers when not in use, turning off computer hard drives and monitors each night, and turning off air-conditioners when not in use. The intervention consisted of three components: (1) posters that showed management support of energy conservation with images of managers modeling the targeted behaviors; (2) communications from various sources within the organization, including top management (e.g., an email from the CEO to introduce the campaign to staff, and an email from the Director of the Information Technology Department to encourage staff to turn off computers overnight); and (3) stickers placed near switches prompting staff to “Turn It Off.” Thus, the campaign clearly emphasized top management support for energy conservation behavior and used prompts to remind employees at the point where the behavior occurred (Abrahamse et al., 2005). A total of 250 posters were displayed in prominent areas (e.g., notice boards) throughout the buildings, and 5,000 stickers were placed immediately next to light switches and power points.

### Measures

In this study we measured our dependent variable, energy use behavior, using both observed and self-reported measures. The independent variables were those we aimed to affect through the intervention and these included perceived top management commitment, organizational culture, descriptive norms, subjective norms, knowledge, and attitudes.

#### Observed Energy Use Behavior

The purpose of the energy audits was to obtain an objective measure of workplace energy behavior, which was the dependent variable for the study. This was measured as the number of energy using appliances left on outside normal office hours. The audits involved a small team of researchers visually inspecting 246 offices located in three different buildings. Some offices were shared/open plan and others were individual. The audits focused on appliances that were targeted in the Turn It Off campaign (lights, computer monitors and hard-drives, chargers, and air-conditioners). For each office the total number of appliances was counted as well as the number of appliances left on, so that the proportion of appliances left on could be calculated (the proportion takes into account any changes in the number of each appliance across the 6 months). The first audit was conducted 2 weeks prior to the intervention, the second audit was conducted 1 month after the intervention, and the third audit was conducted 6 months after the intervention. All three audits were conducted during the evening (outside normal office hours) and employees were not made aware that the audits were being conducted. It was important that employees remained unaware that the audits were being conducted to ensure the baseline results particularly were reflective of current practice. The audits were therefore conducted prior to the distribution of the baseline survey and prior to any communications in relation to the intervention.

#### Self-Reported Energy Use

To complement the observation data, pre- and post-test self-reported behavioral surveys were also conducted. The degree to which participants had engaged in energy conservation behaviors in the week prior to each survey was assessed using four-items, e.g., “please indicate how often in the last week you do the following while at work…turn off lights in unoccupied rooms.” The items were measured on a five-point Likert scale (1 = rarely or never, 5 = always or almost always). The four self-reported behavior items did not form a reliable scale, α = 0.47, and therefore were analyzed individually.

#### Perceived Top Management Commitment

The degree to which participants felt that top management were supportive of energy conservation behavior was measured on a three-items scale adapted from Banerjee et al. (2003), e.g., “Our organization’s energy saving efforts receive full support from our top management.” The items were measured on a five-point Likert scale (1 = strongly disagree, 5 = strongly agree). The scale had an acceptable level of internal reliability, α = 0.84 (Nunnaly, 1978; Tabachnick and Fidell, 2001).

#### Organizational Culture

The degree to which participants felt that the organization’s culture encompassed energy conservation was measured on a four-items scale of internal environmental orientation also adapted from Banerjee et al. (2003), e.g., “energy conservation is a high priority activity in our organization.” The items were measured on a five-point Likert scale (1 = strongly disagree, 5 = strongly agree). The scale had an acceptable level of internal reliability, α = 0.82 (Nunnaly, 1978; Tabachnick and Fidell, 2001).

#### Descriptive Norms, Subjective Norms, and Knowledge

Participants’ perception of descriptive norms regarding energy conservation in the workplace was measured using one-item, “Most staff save energy in the workplace.” Participants’ perception of subjective norms regarding energy conservation in the workplace was measured using one-item, “It is expected of me that I save energy in my workplace.” Finally, participants’ knowledge of how to conserve energy in the workplace was measured with one-item, “I know how to save energy in the workplace.” All three of these items were measured on the same five-point Likert scale (1 = strongly disagree, 5 = strongly agree). The use of single item measures has been shown to be appropriate in previous research on conservation behaviors (Fielding et al., 2012).

#### Attitudes toward Workplace Energy Conservation

Participants’ attitudes toward workplace energy conservation were measured on a three-items scale in accordance with recommendations of Ajzen (1991), e.g., “I think engaging in energy saving behaviors is….” The three-items were measured on different five-point Likert scales (1 = bad, 5 = good; 1 = unimportant, 5 = important; 1 = worthless, 5 = valuable). The scale had an acceptable level of internal reliability, α = 0.81 (Nunnaly, 1978; Tabachnick and Fidell, 2001).

All survey items were administered twice throughout the project. The baseline measurement occurred 1 week prior to the intervention, and the follow-up measurement occurred 1 month after the intervention. Both surveys were conducted *after* the energy audits at the baseline and follow-up periods to avoid any change in behavior that may have occurred because of the topic of the survey.

## Results

### Participant Demographics and Response Rate

The baseline and follow-up surveys were sent to 816 non-clinical staff at a large Australian hospital. A total of 312 staff responded to the baseline survey, and 278 staff responded to the follow-up survey. There were 115 matching responses (14% response rate) across the two surveys. The human resources division of the participant organization confirmed that the matched respondent sample approximated the demographic profile of administrative staff in the organization. We also tested for differences between respondents who completed both surveys and respondents who completed only one of the two surveys and found no significant differences in demographic characteristics, behavior or attitudes between these groups. All analyses reported in relation to the survey and demographics refer to the 115 matching participants. The average age of participants was 40.5 years, and ranged from 17 to 70 years. Approximately two-thirds of participants were female, and one-third were male. Most were employed full-time (87%), some part-time (12%), and very few casuals or other employment types (1%). The average tenure at the organization was 6 years.

### Observed Energy Use Behavior

**FIGURE 2:**
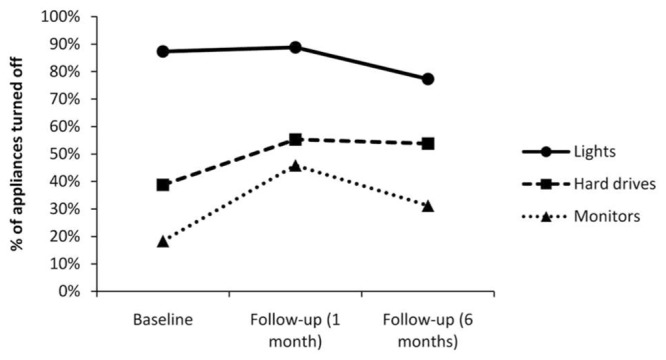
**Findings from the energy audits**.

Energy audits were conducted to provide an observed measure of behavior before the intervention, 1 month after the intervention and 6 months after the intervention. **Figure 2** depicts the key findings from the energy audits. It should be noted that the energy audit results for chargers and air-conditioners are not reported here. The reason for this is that most (more than 90%) of the air-conditioners were centrally controlled (turn on and off automatically), and the number of chargers was too small to carry out meaningful statistical analyses.

#### Lights

At the time of the first audit, 970 out of 1,111 lights were turned off (87.3%). At the time of the second audit, 914 out of 1,029 lights were turned off (88.8%), representing a slight increase (1.5%) in the proportion of lights turned off. However, this difference was not significant, χ*^2^* = 1.16, *ns*. At the time of the third audit, 823 out of 1,064 lights were turned off (77.3%), representing a decrease in the proportion of lights turned off compared to both the first and second audits (10 and 11.5%, respectively). Both differences were significant, χ*^2^* = 37.23, *p* < 0.001 (first vs. third), and χ*^2^* = 48.79, *p* < 0.001 (second vs. third).

#### Hard Drives

At the time of the first audit, 177 out of 464 computer hard drives were turned off (38.8%). At the time of the second audit, 240 out of 434 computer hard drives were turned off (55.3%); representing a 16.5% increase in the proportion of computer hard drives turned off. This difference was significant, χ*^2^* = 26.53, *p* < 0.001. At the time of the third audit, 198 out of 368 computer hard drives were turned off (53.8%), representing a 15% increase in the proportion of computer hard drives turned off compared to the first audit. This difference was also significant, χ*^2^* = 19.63, *p* < 0.001. The difference between the second and third audits was not significant, χ*^2^* = 0.25, *ns*.

#### Monitors

At the time of the first audit, 122 out of 668 monitors were turned off (18.3%). At the time of the second audit, 266 out of 579 monitors were turned off (45.9%), representing a 27.6% increase in the proportion of monitors turned off. This difference was significant, χ*^2^* = 110.86, *p* < 0.001. At the time of the third audit, 137 out of 439 monitors were turned off (31.2%), representing a 12.9% increase in the proportion of monitors turned off compared to the first audit. This difference was significant, χ*^2^* = 24.76, *p* < 0.001. The difference between the second and third audits was also significant, χ*^2^* = 22.66, *p* < 0.001.

### Survey Results

**FIGURE 3:**
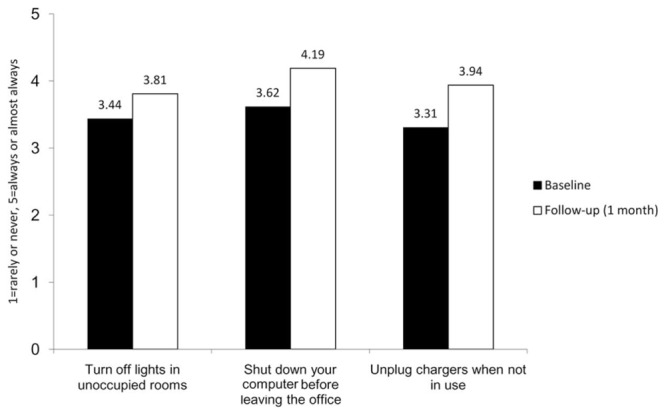
**Changes in self-reported energy conservation behaviors**.

A summary of the mean, standard deviation, and correlation of the survey results at Times 1 and 2 are presented in **Table 1**. Additionally, **Figure 3** depicts the changes in self-reported energy conservation behavior before and after the Turn It Off campaign. The difference in the mean score on the first item, “turn off lights in unoccupied rooms” between baseline and follow-up was significant, *t*(111) = -3.17, *p* = 0.002, such that participants reported turning off lights more often after the Turn It Off campaign (*M* = 3.81) compared to before (*M* = 3.44). The eta squared statistic (η*^2^* = 0.08) indicated a moderate effect size. The difference in the mean score on the second item, “shut down your computer before leaving the office” between baseline and follow-up was significant, *t*(111) = -4.40, *p* < 0.001, such that participants reported turning off computers more often after the Turn It Off campaign (*M* = 4.19) compared to before (*M* = 3.62). The eta squared statistic (η*^2^* = 0.15) indicated a large effect size. The difference in the mean score on the third item “unplug chargers when not in use” between baseline and follow-up was significant, *t*(111) = -4.63, *p* < 0.001, such that participants reported unplugging chargers when not in use more often after the Turn It Off campaign (*M* = 3.94) compared to before (*M* = 3.31). The eta squared statistic (η*^2^* = 0.16) indicated a large effect size. Finally, the difference between scores on the fourth item “turn off air-conditioners when leaving the office” between baseline and follow-up was significant, *t*(52) = -2.13, *p* = 0.038, such that participants reported turning off air-conditioners more often after the Turn It Off campaign (*M* = 3.64) compared to before (*M* = 3.26). The eta squared statistic (η*^2^* = 0.08) indicated a moderate effect size.

**Table 1:** Mean, standard deviation, and bivariate correlations among self-reported variables^a^.

	Mean	*SD*	1	2	3	4	5	6	7	8	9	10
(1) Lights	3.45	1.42	–	0.15	0.14	0.67^∗∗^	0.16	0.18	0.23^∗^	0.17	0.34^∗∗^	0.28^∗∗^
(2) Computers	3.62	1.54	0.13	–	0.13	0.18	0.15	0.13	-0.01	0.19^∗^	0.17	0.24^∗^
(3) Monitors	3.31	1.63	0.18	0.16	–	0.19	0.12	0.09	0.04	0.15	0.33^∗∗^	0.30^∗∗^
(4) Air conditioners	2.55	1.83	0.37^∗∗^	0.13	0.13	–	0.33^∗^	0.43^∗∗^	0.04	0.36^∗∗^	0.38^∗∗^	0.31^∗^
(5) Top Mgmt Commitment	3.29	0.72	0.15	0.00	0.10	0.24^∗^	**0.84**	0.84^∗∗^	0.16	0.52^∗∗^	0.68^∗∗^	0.22^∗^
(6) Organizational Culture	2.94	0.74	0.26^∗∗^	-0.01	0.18	0.25^∗∗^	0.73^∗∗^	**0.82**	0.18	0.58^∗∗^	0.66^∗∗^	0.18
(7) Attitudes	4.65	0.48	0.19	0.12	0.15	0.21^∗^	-0.10	-0.10	**0.81**	0.06	0.17	0.23^∗^
(8) Descriptive Norms	2.69	0.83	0.27^∗∗^	-0.01	0.19^∗^	0.24^∗^	0.34^∗∗^	0.55^∗∗^	-0.11		0.43^∗∗^	0.11
(9) Subjective Norms	3.31	0.95	0.24^∗^	0.13	0.19^∗^	0.29^∗∗^	0.51^∗∗^	0.53^∗∗^	-0.07	0.47^∗∗^		0.43^∗∗^
(10) Knowledge	3.87	0.79	0.33^∗∗^	0.17	0.27^∗∗^	0.25^∗∗^	0.12	0.24^∗^	0.18	0.14	0.31^∗∗^	

*^a^Mean and SD are at Time 1. Time 1 correlations are presented below the diagonal, Time 2 correlations are presented above the diagonal. Cronbach’s alpha for computed subscales are in bold on the diagonal. Asterisk’s represent the following: ^∗^p < 0.05, ^∗∗^p < 0.01.*

**FIGURE 4:**
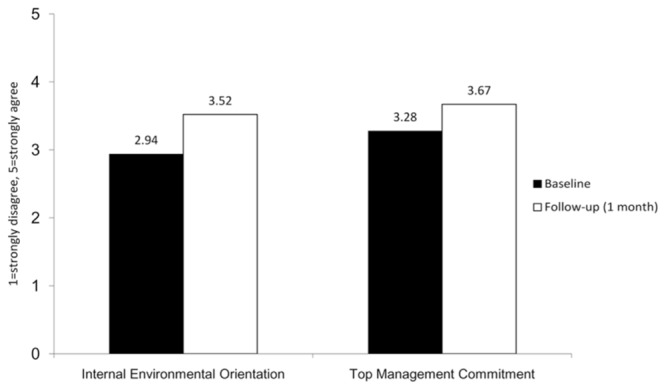
**Changes in corporate environmental climate**.

**Figure 4** depicts the changes in perceived top management commitment and internal environmental orientation in the organization before and after the Turn It Off campaign. The difference in perceived top management commitment between baseline and follow-up was significant, *t*(107) = -5.66, *p* < 0.001. Participants reported stronger top management commitment after the Turn It Off campaign (*M* = 3.68) compared to before (*M* = 3.28). The eta squared statistic (η*^2^* = 0.23) indicated a large effect size. Similarly, the difference in perceived internal environmental orientation between baseline and follow-up was also significant, *t*(107) = -7.66, *p* < 0.001. Participants perceived a more positive internal environmental orientation after the Turn It Off campaign (*M* = 3.52) compared to before (*M* = 2.94). The eta squared statistic (η*^2^* = 0.35) indicated a large effect size.

**FIGURE 5:**
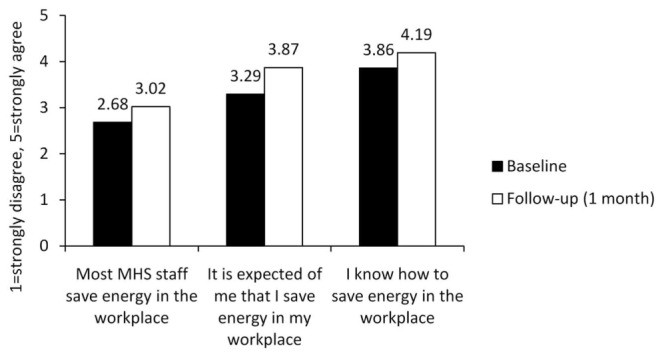
**Changes in norms and knowledge of energy conservation behavior**.

The difference in descriptive norms between baseline and follow-up was significant, *t*(106) = -3.91, *p* < 0.001. Participants perceived that more staff saved energy in the workplace after the Turn It Off campaign (*M* = 3.02) compared to before (*M* = 2.68). The eta squared statistic (η*^2^* = 0.13) indicated a moderate effect size. The difference in subjective norms between baseline and follow-up was significant, *t*(106) = -5.63, *p* < 0.001. Participants perceived greater expectations of energy conservation after the Turn It Off campaign (*M* = 3.87) compared to before (*M* = 3.29). The eta squared statistic (η*^2^* = 0.23) indicated a large effect size. The difference in knowledge regarding energy conservation between baseline and follow-up was significant, *t*(105) = -4.77, *p* < 0.001. Participants reported greater knowledge regarding how to save energy in the workplace after the Turn It Off campaign (*M* = 4.19) compared to before (*M* = 3.86). The eta squared statistic (η*^2^* = 0.18) indicated a large effect size. Finally, attitudes toward workplace energy conservation did not significantly change throughout the study, *t*(101) = -0.68, *p* = 0.495. The mean before the Turn It Off campaign was 4.65, compared to 4.68 after the campaign. **Figure 5** depicts the changes in norms and knowledge about energy conservation behavior before and after the Turn It Off campaign.

## Discussion and Conclusion

Our aim in this research was to examine the efficacy of an intervention to reduce energy use in a workplace context. We did this by designing an intervention that used the influence of top management commitment on energy conservation in order to embed the behavior over the long-term. Our study extends past research by demonstrating that the use of influence strategies in combination with prompts is an effective strategy to encourage energy conservation behavior in the workplace. Although past research has shown that prompts are effective in household settings, the application to the workplace had not been empirically tested (Schultz et al., 1995; Lehman and Geller, 2004; Osbaldiston, 2004; Osbaldiston and Schott, 2012). Our results extend previous research by showing that these types of interventions may be effective only for particular behaviors. Indeed, our intervention was particularly successful for behaviors with a strong element of individual responsibility, such as turning off computer monitors and hard drives. The intervention was not successful in changing behaviors that are more collectively oriented such as turning off lights. Further, the current study has successfully addressed two limitations of previous research: (1) the use of self-reported measures as an indicator of behavior; and (2) the cross-sectional nature of studies of top management commitment (Ramus and Steger, 2000; Robertson and Barling, 2013).

Survey results demonstrated that the energy conservation intervention led to positive changes in self-reported energy conservation behavior, perceived top management commitment, perceived internal environmental orientation, subjective norms, descriptive norms, and knowledge of energy conservation behavior. There were, however, no changes in attitudes. It is possible that this may be a result of a ceiling effect (attitudes toward energy conservation behavior were quite high at baseline and remained high at follow-up). However, these results provide empirical support for the assertions by Young et al. (2015) that it is not necessary to change attitudes in order to effectively change behavior.

The results of the energy audits were mixed. For computer monitors and hard drives the results showed that the intervention was effective at increasing the proportion of appliances turned off, and these findings were maintained at a 6-months follow-up. However, for lights the results were not as consistent – in fact, the reverse effect was found.

One possible explanation for the mixed energy audit findings could be that computer hard drives and monitors are behaviors that have a strong element of individual responsibility, whereas lights are often shared resources and thus there is a diffusion of responsibility for those appliances (particularly in shared or open plan offices). Story and Forsyth (2008) argued that responsibility is an antecedent to behavioral and contribution intentions, and that awareness (both directly and indirectly through appraisal) of an issue leads to a sense of personal responsibility for that issue. It is possible that by making individual responsibility more salient in the intervention, that participants focused more on this to the detriment of the more collective behavior of switching off lights. Indeed, as shown in **Figure 2**, the percentage of lights that were turned off decreased over the course of the research.

Future research in this area should examine the role of responsibility for different appliances, and compare the effectiveness of interventions for individual offices vs. shared/open plan offices. Our intervention worked well for behaviors with an element of individual responsibility, but another approach may be required for collective behaviors or behaviors where there is a diffusion of responsibility. Another alternative explanation for this finding could be that of moral self-licensing (Merritt et al., 2010). It could be, for example, that employees engage in individual behaviors such as turning off computers and monitors and feel they have gained moral credit. When it comes to engaging in shared behaviors such as turning off lights, employees feel they do not have to engage in these behaviors because they have earned moral credits. Similarly, this could be a symptom of social loafing, whereby people exert less effort or have less motivation to achieve a goal when they are working in a group (Simms and Nichols, 2014; Frederiks et al., 2015). It is unclear from this study what mechanism is driving this behavior and this warrants further attention in future research.

As with most field research, there are some limitations that need to be acknowledged. The first limitation is that the design does not enable the disentangling of the effects of top management support and prompts. We can say that the combination of the two intervention types was successful; it is not possible, however, to determine the extent to which prompts were effective as compared to top management support. Future research that examines these intervention strategies separately would be of benefit in determining the specific effectiveness of each approach.

The study utilized a pre-test post-test design. There are other designs that may have shown more conclusive results such as an ABAB design where the intervention is implemented, removed, reinstated, and then removed with measures at each period (see for example, Sussman and Gifford, 2012). An ABAB design could not be utilized in the current study because this design was not consistent with the organization’s goals. The organizational aim was to promote energy conservation behavior and it was therefore not possible to implement and then remove the intervention during the course of the study as would be required by the ABAB design. Furthermore, the organizational constraints meant that we were not able to withhold the intervention from a control group. The use of a control group was not considered to be in line with the organization’s goals, thus the intervention was applied to all staff members. We used the measurements of both self-report and observations pre- and post-intervention and showed a change in behavior, however, the absence of a control group remains a limitation of this research.

Another factor to consider is habituation to prompts. De Young (1993) argued that prompts are ineffective in the long-term, particularly once people become habituated to them. Our research showed that the intervention was successful for individual behaviors after a 6-months period. Although there was some reduction in effectiveness at the 6-months follow-up there remained a significant reduction in energy using behaviors after 6 months as compared to the baseline measurements. However, future research would be valuable to test the effectiveness on a longer time scale. Research has shown that once a prompt is removed, behavior can return to baseline levels (De Young, 1993). Future research would be of benefit to monitor the effectiveness of prompts and whether or not the target audience becomes habituated to them. Strategies to reduce habituation in workplace settings could also be tested. Making a change to the prompt (stimulus specificity) and introducing a new prompt (dishabituation) are two techniques known to reduce habituation to stimuli in lab studies (McSweeney, 2004), but this remains to be tested in workplace settings.

Finally, the role of organizational culture could be explored further. In the current study, internal environmental orientation was measured but not manipulated. Since culture is an important variable in organizational change for sustainability (Russell and McIntosh, 2011; Young et al., 2015), it is likely that the internal environmental orientation of the organization had an effect on participants’ willingness to engage in energy conservation behavior. Future research that examines the effect of the internal environmental orientation of the organization would be of benefit in this area.

In this research we have extended previous research on top management commitment and demonstrated that this type of intervention strategy is an effective way of encouraging energy conservation behaviors in the workplace. Furthermore, we have extended past research by demonstrating how top management commitment can be used to influence behavior over the long-term. Furthermore, the use of prompts in conjunction with top management commitment was shown to lead to positive changes in perceptions of top management commitment and internal environmental orientation. The opportunities for future research in the area of workplace pro-environmental behavior are vast, and include examining the role of responsibility, habituation, and internal environmental orientation.

## Author Contributions

All authors listed, have made substantial, direct and intellectual contribution to the work, and approved it for publication.

## Conflict of Interest Statement

The authors declare that the research was conducted in the absence of any commercial or financial relationships that could be construed as a potential conflict of interest.
